# 2023 Acknowledgment of *Microbiology Spectrum* Reviewing Editors

**DOI:** 10.1128/spectrum.01185-23

**Published:** 2023-04-13

**Authors:** Christina A. Cuomo

**Affiliations:** a Broad Institute of MIT and Harvard, Cambridge, Massachusetts, USA

## EDITORIAL

In April 2022, we launched a program for early-career microbiologists from across the globe to participate in *Microbiology Spectrum* via a new role, the Reviewing Editor. The Reviewing Editor role is distinct from the Editor role in that while Editors choose peer reviewers and make decisions on papers, the Reviewing Editors serve as expert reviewers for one to two papers per month (a maximum of 24 per year) and receive feedback on their reviews. Reviewing Editors will advance to the Editor role on the basis of their performance and subject area expertise needs for Editors. Currently, we have 84 Reviewing Editors from 29 countries with expertise across microbiology. In the past year, our Reviewing Editors have provided over 600 reviews for the journal—extraordinary service for which we are very grateful. We thank all of our Reviewing Editors for their dedication to the journal and its success.

Philips O. Akinwole
Valeria Allizond
Cassio Almeida-da-Silva
Jorge Arca-Suárez
Charina Gracia B. Banaay
Arden Baylink
Michael G. Becker
Irene Burckhardt
Ana Cabrera
Amanda E. Calvert
Siu-Kei Chow
Vito Colella
Francesco Comandatore
Bernadette J. Connors
Jôice D. Corrêa
Luciana J. Costa
Maria A. De Francesco
Kanan T. Desai
Bismarck Dinko
Yi-Chin Fan
Hui-Chen Foreman
Estela M. Galvan
Miguel Angel Garcia Bereguiain
Sophia B. Georghiou
Emilia Ghelardi
Laura Huber
Yosef D. Huberman
Kelly Ishida
Juan P. Jaworski
Hyung Jong Jin
Teny M. John
Denis E. Kainov
Johanna J. Kenyon
Katharina Kujala
Yongxin Li
Xiaoyan Lin
Amelia R. Lindsey
Benjamin M. Liu
Sonia Luque
Zhe Lyu
Jose Martinez-Navio
Jun Miao
Sheema Mir
Walaa K. Mousa
Kattia Núñez-Montero
Dhammika H. Navarathna
Jowita Samanta Niczyporuk
Michael Owusu
Elias A. Rahal
Kessendri Reddy
Christopher A. Rice
Antonio Ruzzini
Sanghamitra Saha
Garima Singh
Nicolas Soler
Katie L. Summers
Hao Tan
Fang Tang
Li Tao
Anuraj Theradiyil Sukumaran
Cecilia Thompson
Felix N. Toka
Maria Tomas
Xinzhao Tong
Deeksha Tripathi
Gareth Trubl
Pablo Tsukayama
Filipa F. Vale
Vanessa A. Varaljay
Musturi Venkataramana
Crista B. Wadsworth
Caray A. Walker
Fang Wang
Wei Wang
Yan Wang
Yi Wang
Michael G. Whitfield
Gregory Wiedman
Anne L. Wyllie
Zhu Xiaoyu
Pragya D. Yadav
Srujana Samhita S. Yadavalli
Kayvan Zainabadi
Krzysztof Zawierucha

